# Influence of N^ε^-Lysine Acetylation on the Formation of Protein Aggregates and Antibiotic Persistence in *E. coli*

**DOI:** 10.3390/molecules29020383

**Published:** 2024-01-12

**Authors:** Karolina Stojowska-Swędrzyńska, Dorota Kuczyńska-Wiśnik, Ewa Laskowska

**Affiliations:** Department of General and Medical Biochemistry, Faculty of Biology, University of Gdansk, Wita Stwosza 59, 80-308 Gdansk, Poland; karolina.stojowska-swedrzynska@ug.edu.pl (K.S.-S.); dorota.kuczynska-wisnik@ug.edu.pl (D.K.-W.)

**Keywords:** N^ε^-lysine acetylation, bacterial persisters, protein aggregation

## Abstract

Numerous studies indicate that reversible N^ε^-lysine acetylation in bacteria may play a key role in the regulation of metabolic processes, transcription and translation, biofilm formation, virulence, and drug resistance. Using appropriate mutant strains deficient in non-enzymatic acetylation and enzymatic acetylation or deacetylation pathways, we investigated the influence of protein acetylation on cell viability, protein aggregation, and persister formation in *Escherichia coli*. Lysine acetylation was found to increase protein aggregation and cell viability under the late stationary phase. Moreover, increased lysine acetylation stimulated the formation of persisters. These results suggest that acetylation-dependent aggregation may improve the survival of bacteria under adverse conditions (such as the late stationary phase) and during antibiotic treatment. Further experiments revealed that acetylation-favorable conditions may increase persister formation in *Klebsiella pneumoniae* clinical isolate. However, the exact mechanisms underlying the relationship between acetylation and persistence in this pathogen remain to be elucidated.

## 1. Introduction

**Figure 1:**
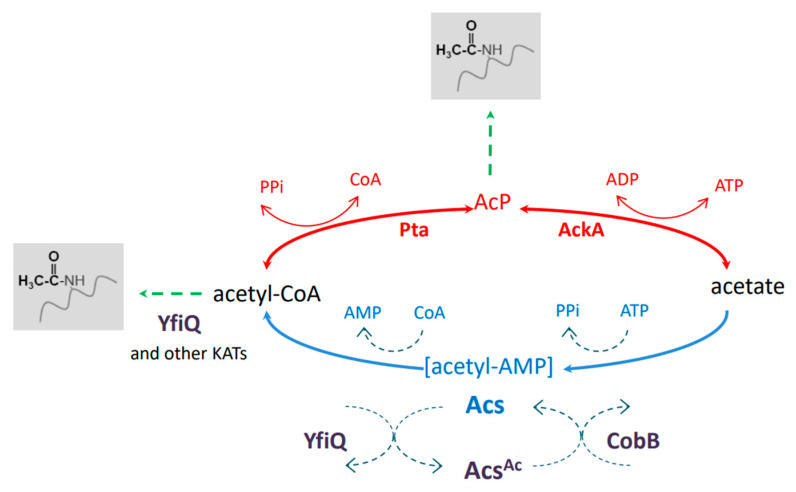
N^ε^-lysine acetylation in *E. coli.* The nonenzymatic AcP-dependent and KAT-dependent pathways are responsible for protein acetylation. YfiQ and CobB participate in the regulation of the acetyl-CoA synthase Acs. See the text for more details. AcP, acetyl phosphate; KATs, lysine acetyltransferases.

Recent studies indicate that reversible lysine acetylation plays important roles in numerous processes in bacteria, including the regulation of metabolism, transcription, translation, stress response, and virulence [1,2,3,4,5,6,7,8,9,10,11]. It has been estimated that up to 40% of all bacterial proteins are modified by lysine acetylation, depending on the species [10]. In *E. coli*, an acetyl-phosphate (AcP)-dependent nonenzymatic reaction is the main mechanism of protein acetylation. AcP is synthesized from acetyl-CoA or acetate by the phosphotransacetylase Pta or acetate kinase AckA, respectively (Figure 1). Both enzymes, Pta and AckA, catalyze reverse reactions; thus, acetyl-CoA, acetate phosphate, and acetate are mutually convertible. Apart from nonenzymatic acetylation by AcP, the lysine acetyltransferases (KATs) PatZ, RimI, YjaB, YiaC, and PhnO modify *E. coli* proteins by transferring acetyl groups from acetyl-CoA to N^ε^-lysine residues [12].

Among the known *E. coli* acetyltransferases, YfiQ (PatZ) has been shown to modify most of the acetylated proteins detected upon overexpression of the selected KATs. AcP- and KAT-dependent acetylation differ in their substrates: AcP modifies mainly the central metabolic enzymes, whereas KATs recognize enzymes involved in the branch points of central metabolism [12]. N^ε^-lysine acetylation is reversed by the deacetylase CobB, which belongs to the NAD^+^-dependent sirtuin family [13]. It has been demonstrated that CobB can remove acetyl groups regardless of the acetyl donor or acetylation mechanism [14]. Moreover, CobB has been found to reverse other acyl modifications (succinylation and 2-hydroxyisobutyrylation) [15,16]. It has been suggested that YcgC is another *E. coli* deacetylase, with a distinct set of substrates from CobB and an NAD^+^-independent mode of action [17]. However, a recent report indicated that YcgC has no deacetylase activity [18].

Since acetyltransferases use acetyl-CoA for acetylation and deacetylases use NAD+ as a co-substrate for deacetylation, acetylation strongly depends on the metabolic status of the cell. On the other hand, the activities of numerous metabolic enzymes are affected by acetylation, especially those involved in synthesizing or utilizing acetyl-CoA [10]. One of the best-characterized key enzymes regulated by acetylation in eukaryotes and bacteria—including *E. coli*, *Salmonella enterica*, *Bacillus subtilis*, and *Mycobacterium tuberculosis*—is acetyl-CoA synthetase (Acs) [19,20,21,22]. Acs synthesizes acetyl-CoA in two steps: (1) adenylation, in which an acetyl-AMP intermediate is formed; (2) replacement of AMP by CoA to form acetyl-CoA. Acs is regulated at the transcriptional and post-translational levels [22]. Upon entry into the stationary phase, cAMP upregulates the expression of the *acs* and *yfiQ* genes. Acetylation of Acs by YfiQ restricts the activity of the synthetase to prevent AMP accumulation and growth arrest. Acs resumes its activity after deacetylation by CobB [22].

Recent studies suggest that the regulatory role of acetylation in bacteria is overestimated [23]. It has been shown that global lysine acetylation results from the acetate overflow and targets accessible lysine residues rather than specific enzymes. Growth in excess glucose or other sugars may lead to an imbalance between the production and assimilation of acetyl-CoA, the main acetate precursor. Acetate overflow is accompanied by the overproduction of acetyl phosphate (AcP) [9].

N^ε^-lysine acetylation neutralizes the positively charged lysine side chain and increases its size and hydrophobicity, which may impact protein–protein and protein–nucleic acid interactions, leading to the formation of protein aggregates. Our previous studies have revealed that protein acetylation may affect the solubility and biological activity of a model recombinant protein VP1GFP, which tends to form inclusion bodies (IBs) [24]. The formation of endogenous protein aggregates during the late stationary phase in *E. coli* cells was also influenced by acetylation. In general, decreased acetylation (in Δ*ackA-pta* cells) postponed the formation of endogenous protein aggregates and IBs, whereas decreased deacetylation (due to the lack of CobB) enhanced aggregation. Non-acetylated IBs had significantly higher biological activity than their acetylated counterparts [24]. It is important to note that after acetylation in vitro, several eukaryotic proteins form aggregates, while others are stabilized in a soluble form. The influence of acetylation on the tendency to form aggregates is most likely determined by the location of the acetylation sites and their functional implications [25,26,27].

Loss of function and toxicity are often associated with protein aggregation. However, a growing body of evidence has shown that protein aggregation may exert beneficial effects in bacteria exposed to different stressful conditions [28,29,30,31,32,33]. First, mature aggregates are assumed to be less toxic than their soluble, misfolded, and oligomeric precursors, which can interact nonspecifically with other macromolecules and membranes [32]. Second, the aggregates may play the role of compartments that preserve and protect proteins against irreversible inactivation or degradation. For example, it has been demonstrated that the formation of protein aggregates enabled *Acinetobacter baumannii* to survive desiccation stress [33]. It has also been found that a model enzyme β-galactosidase sequestered in the *A. baumannii* aggregates retained its activity. Aggregates or condensates that contribute to different regulatory and protective mechanisms are often formed via liquid—liquid phase separation [34,35,36]. These so-called membrane-less organelles (MLOs) include the ParABS system required for proper plasmid and chromosome segregation during cell division, RNA polymerase clusters, Dps-nucleic acid condensates, and aggresomes containing hundreds of different proteins [30,37,38,39].

Numerous studies indicate that protein aggregation and acetylation may be responsible for antibiotic resistance or tolerance in *E. coli* and other Gram-negative bacteria [11,29,30,40,41,42]. One example is the correlation between the aggregation of endogenous *E. coli* proteins and persisters. Persisters are dormant cells, usually constituting a small part of the bacterial population that can withstand high concentrations of antibiotics. In contrast to resistant mutants, persisters are phenotypic variants of wild-type bacteria; after antibiotic treatment, persisters may resume growth, becoming drug-sensitive again [43,44]. The *E. coli* aggregates contain EF-Tu and other essential proteins participating in different processes. Their sequestration in aggregates may inhibit translation and metabolic processes, leading to a dormant state. It has been proposed that the main cause of protein aggregation is ATP depletion. ATP level reduction diminishes the efficiency of ATP-dependent molecular chaperones and proteases responsible for removing damaged and aggregated proteins. Furthermore, persister resuscitation requires removing aggregates via ATP-dependent molecular chaperones DnaK and ClpB [41].

Recently, a mechanism of antibiotic resistance by acetylation in *E. coli* has been reported [42]. The comparison of acetylated proteomes from wild-type and antibiotic-resistant *E. coli* strains showed that the key enzymes in various pathways were differentially acetylated. Generally, protein acetylation negatively regulated metabolism in antibiotic-resistant strains. In particular, the acetylation of K_413_ in pyruvate kinase PykF, the pyruvate cycle enzyme, inhibited its activity and slowed down the TCA cycle, resulting in a low-energy metabolism and resistance to antibiotics. Deacetylation of PykF increased the energy metabolism and restored antibiotic sensitivity. It is worth noting that different bacterial toxins encoded by the toxin-antitoxin systems have been identified as acetyltransferases contributing to persister formation. AtaT from enterohemorrhagic *E. coli*, TacT from *S. typhimurium*, and KacT from *K. pneumoniae* transfer the acetyl group from acetyl-CoA to the amine group of aminoacyl-tRNA molecules, leading to the inhibition of translation and dormancy [45,46,47].

In summary, these reports strongly suggest that protein acetylation and aggregation may improve the survival of bacteria under unfavorable conditions, including antibiotic treatment. This study aims to investigate the influence of two main protein acetylation/deacetylation pathways—nonenzymatic Pta-AckA and enzymatic YfiQ-dependent acetylation—on cell viability, protein aggregation, and persister formation in *E. coli*.

## 2. Results

### 2.1. Lysine Acetylation Enhances Protein Aggregation and E. coli Viability under Late Stationary Phase

Aggregates formed in *E. coli* under the stationary phase contain over 600 proteins, including ribosomal proteins and enzymes involved in glycolysis, the TCA cycle, and fatty acid synthesis [48]. DNA-binding protein from starved cells (Dps) is the most abundant component of the aggregate protein. Dps undergoes co-crystalization with DNA and thereby cosediments with insoluble protein. Dps and multiple other components of the aggregates were identified as proteins prone to liquid–liquid phase separation [34]. To analyze the influence of lysine acetylation on the formation of protein aggregates, we used different *E. coli* mutants deficient in enzymatic or nonenzymatic acetylation and deacetylation pathways (Table 1).

**Figure 2:**
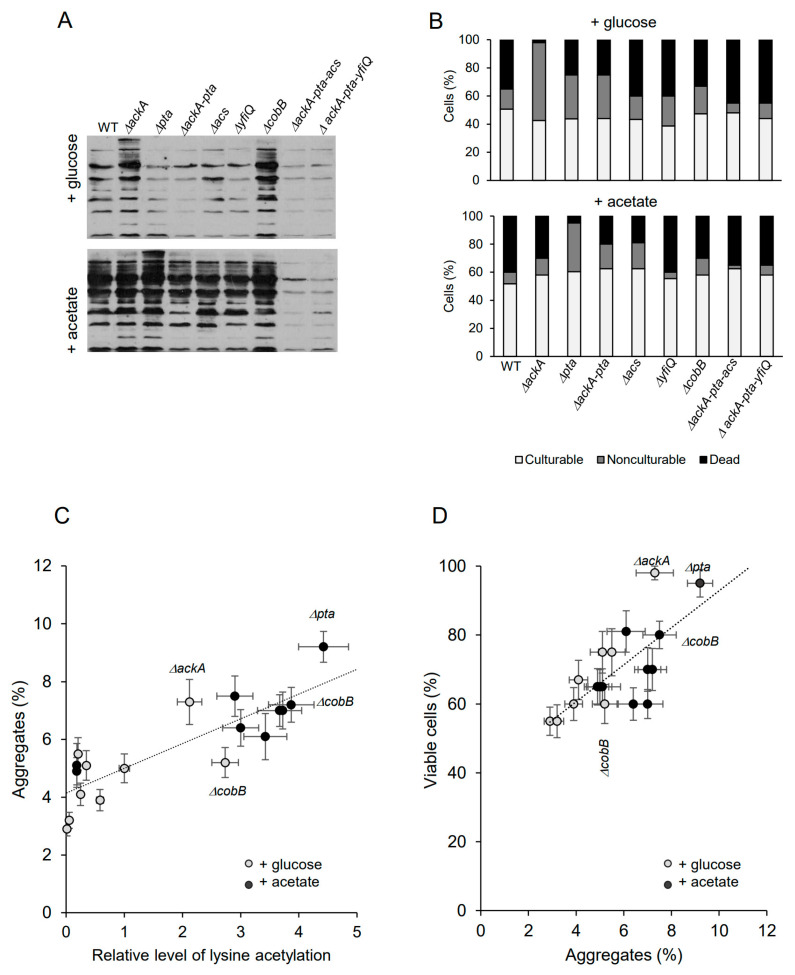
Protein aggregation and persister formation are correlated to lysine acetylation. (**A**) Protein acetylation in whole *E. coli* extracts. The cultures were supplemented with 0.4% glucose or 25 mM acetate. Acetyl-lysine was immunodetected as described in the Section 4. Samples corresponding to the same number of cells were loaded on gels. Representative results are shown. (**B**) Percentage of culturable, nonculturable, and dead bacteria in 48 h cultures supplemented with 0.4% glucose or 25 mM acetate. The total cell number reached ~1.6 × 10^7^ (WT, Δ*ackA*, Δ*pta*, Δ*ackA-pta*, Δ*yfiQ*, and Δ*cobB*) and 1.4 × 10^7^ (Δ*ackA-pta-acs* and Δ*ackA-pta-yfiQ*). (**C**) Correlation between protein acetylation and aggregation. Relative levels of acetylated proteins were estimated by densitometry. Protein aggregates were isolated and estimated as described in the Section 4. (**D**) Correlation between protein aggregation and cell viability. Error bars represent the standard deviation of three independent experiments. Data presented in panels (**C**,**D**) are summarized in Supplementary Table S1.

Bacteria have been grown under acetylation-favorable conditions in TB7 minimal media supplemented with 0.4% glucose or 25 mM sodium acetate until the late stationary phase (48 h). Antibodies against acetyl-lysine were used to detect acetylated proteins (Figure 2A). In the glucose-supplemented cultures (Figure 2A), the level of acetylated proteins increased significantly in Δ*ackA* cells (accumulating excess AcP) and decreased in the Δ*ackA-pta* mutant (not producing AcP) and triple mutants deficient in acetyl-CoA and AcP. The lack of CobB deacetylase resulted in enhanced protein acetylation. These results were consistent with previous studies reported by Kuhn et al. and Schilling et al. [23,49]. In the presence of acetate (Figure 2A), the signal was significantly enhanced in all tested strains (except the Δ*ackA-pta-acs* and Δ*ackA-pta-yfiQ* strains) compared to cultures supplemented with glucose. Depending on the mutation, different acetylation pathways were activated and acetate was converted into AcP and/or acetyl-CoA. The highest level of acetylated proteins was detected in Δ*pta* cells (which accumulated AcP) and in the CobB deacetylase deficient strain. All strains showed decreased culturability after 48 h of growth (Figure 2B; 40–50% and 50–60% of the total cell number in glucose and acetate-supplemented cultures, respectively). Significant differences were observed in the level of nonculturable and dead bacteria. Increased numbers of nonculturable bacteria were detected in the strains overproducing AcP: Δ*ackA* grown in the presence of glucose (55% of the total cell number) and *pta* cells from the acetate-supplemented culture (35% of the total cell number). There was a weak correlation between the levels of acetylation and cell viability (Spearman’s coefficient of 0.37). It should be noted that the mutations may cause pleiotropic effects which are not directly reflected by the intensity of bulk acetylation of proteins [50]. Nevertheless, enhanced protein acetylation strongly correlated with increased protein aggregation (Figure 2C, Spearman’s coefficient of 0.83), in agreement with the previous study [24,48]. Acetate generally induced higher protein aggregation than glucose. Interestingly, the highest level of aggregated proteins was detected in strains that produced the highest number of viable cells (Figure 2D): Δ*pta* in acetate-supplemented LB (9.2% of aggregated proteins, 95% of viable cells) and Δ*ackA* in glucose-supplemented LB (7.3% of aggregated proteins, 98% of viable cells). This confirms that protein aggregates may play protective functions under a prolonged stationary phase [30]. The Spearman’s rank correlation coefficient between the level of protein aggregates and cell viability was 0.68.

### 2.2. Lysine Acetylation Stimulates the Formation of Persisters in E. coli

**Figure 3:**
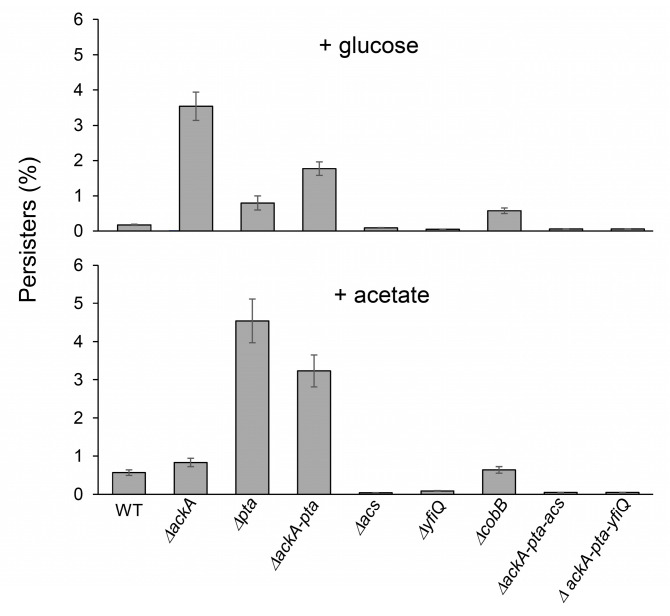
Persister levels in *E. coli* strains with impaired acetylation/deacetylation pathways. The percentage of persisters was estimated as described in the Section 4 (100% = CFU before antibiotic treatment). Error bars represent the standard deviation of three independent experiments.

To answer the question of whether protein acetylation and aggregation may affect persister formation [40,41,42], we next exposed the tested *E. coli* strains to ofloxacin treatment. Subpopulations that survive antibiotic treatment are heterogeneous and may contain “shallow” and “deep” persisters and nonculturable bacteria with different abilities to resume growth on LA plates [51,52,53]. Using live/dead staining, we found that after antibiotic exposure, most cultures contained dead and nonculturable bacteria, whereas persisters represented a small fraction of the population (Table 2). Notably, increased levels of nonculturable bacteria and persisters (Table 2, Figure 3) were detected in the Δ*ackA* (+glucose) and Δ*pta* (+acetate) strains characterized by enhanced acetylation. A moderate positive correlation was detected between the acetylation levels and persisters (Spearman’s coefficient of 0.46). These results indicated that protein acetylation and aggregation can increase bacterial survival and promote persisters’ formation.

### 2.3. Protein Acetylation and Persister Formation in Clinical Isolates of Gram-Negative and Gram-Positive Bacteria

**Figure 4:**
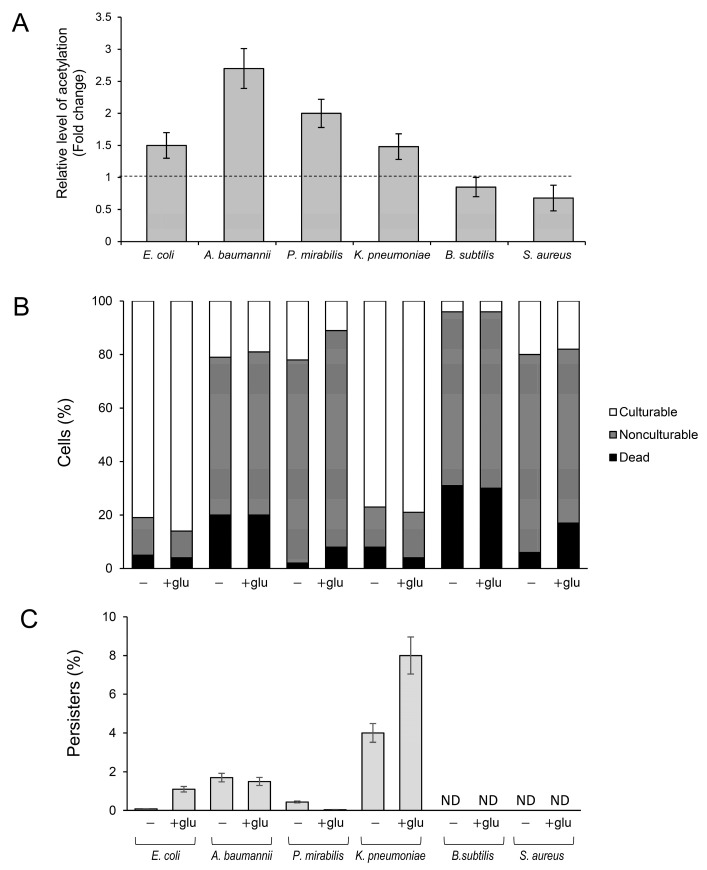
Protein acetylation and the formation of persisters in Gram-negative and Gram-positive clinical isolates. (**A**) Protein acetylation level in bacterial extracts. The cultures were grown at 37 °C in LB medium with or without 0.4% glucose. After 24 h, the samples were collected, and acetyl-lysine was immunodetected, as described in the Section 4. Relative levels of acetylated proteins were estimated via densitometry using the ImageJ version 1.53k software. The results are presented as a ratio between acetyl lysine levels in LB + glucose and LB cultures. (**B**) Percentage of culturable, nonculturable, and dead bacteria. (**C**) Persister levels, expressed as a percentage of CFU before antibiotic treatment, were estimated as described in the Section 4. The 24 h cultures were diluted 1:100 in fresh LB or LB +0.4% glucose medium supplemented with ofloxacin (50 × MIC, *E. coli*) or meropenem (50 × MIC, *A. baumannii*, *K. pneumoniae*, and *P. mirabilis*). Error bars represent the standard deviation of three independent experiments.

Antibiotic-tolerant persisters are a serious medical problem and the main cause of recurrent infections [29,44]. Therefore, to further analyze the link between the frequency of persisters and lysine acetylation, we used selected clinical isolates of Gram-negative (*K. pneumonia*, *A. baumannii*, *P. vulgaris)* and Gram-positive bacteria (*S. aureus* and *B. subtilis*). Instead of using acetylation/deacetylation-deficient mutants (which were not available for all bacterial species), we used different media and growth conditions to induce lysine acetylation in the clinical isolates and *E. coli* BW25113 as the control strain (Figure 4A). Most strains grew efficiently in an LB medium supplemented with glucose, reaching the late stationary phase with less than 20% of dead cells in the entire culture population (Figure 4B). Significant growth inhibition or less-reproducible results were obtained with other media (such as TB7 or Mueller–Hinton broth) or at growth temperatures lower than 37 °C. Upon growth in the presence of 0.4% glucose, significantly increased acetylation was observed in the control *E. coli* strain and Gram-negative clinical isolates; meanwhile, in Gram-positive bacteria, acetylation levels remained almost unchanged compared to the medium without glucose (Figure 4A). Except for *E. coli* and *K. pneumoniae* cultures, all strains lost their culturability independently of the presence of glucose in the medium. Acetylation-favorable conditions slightly (but statistically significantly, *p* < 0.05) increased the culturability of *E. coli* (from ~81 to 86% of the total cell number) and reduced the number of dead cells in *K. pneumoniae* (from ~8 to 2% of the total number of cells) (Figure 4B).

The highest number of persisters induced by acetylation was detected in *K. pneumoniae* cultures: ~4% and ~8% in LB and LB + glucose medium, respectively (Figure 4C). A significantly increased frequency of persisters under acetylation-favorable conditions was also observed in *E. coli* (0.08% and 1.1% in LB and LB + glucose medium, respectively). In other tested strains, glucose supplementation did not affect (*A. baumannii)* or decreased (*P. mirabilis)* the level of persisters. In summary, these results suggest that the relation between lysine acetylation, viability, and persister formation might be restricted only to the selected members of *Enterobacteriaceae* (*E. coli* and *K. pneumoniae*). Further studies are needed to confirm this assumption and explain the mechanism linking acetylation with persistence.

## 3. Discussion

Protein acetylation in bacteria may serve many functions, including regulation of central metabolism, growth control, virulence, and biofilm formation [1,46,54,55,56,57]. On the global level, acetylation may increase stress resistance [42,58], prevent excessive carbon flux, or serve as a storage mechanism for carbon subunits in the cell [9]. Acetylation may also protect proteins against irreversible detrimental modifications such as carbonylation or glycation, since all these modifications may occur on the lysine residue [9,59]. Similar protection mechanisms against glycation have been reported in eukaryotic cells [60,61]. Accumulation of glycation products is often attributed to aging, diabetes, and other pathological conditions in humans. For example, Zheng et al. demonstrated that lysine acetylation protects histones from enhanced cancer-related glycation of proteins in breast cancer cell lines [60]. The results presented in this study suggest that lysine acetylation in *E. coli* may play an additional protective function by promoting the formation of protein aggregates. We found that those *E. coli* strains that accumulated enhanced levels of acetylated protein aggregates showed increased survival under a prolonged stationary phase in the presence of glucose or acetate (Figure 2). As mentioned, the aggregates may preserve and protect proteins against irreversible inactivation or degradation and slow down metabolism by trapping essential proteins, leading to dormancy. Consequently, enhanced protein acetylation may increase persister formation (Figure 3). Further studies are needed to better understand the link between acetylation/aggregation and persistence. It is possible that acetylation of a particular soluble protein(s), rather than aggregates, triggers persister formation. It would also be interesting to investigate whether deacetylation is a prerequisite for aggregate solubilization and for awakening persisters from a dormant state. These questions are particularly important in the case of clinical pathogens.

The mechanisms responsible for lysine acetylation and the processes regulated by acetylation in pathogens are becoming better understood. It has been demonstrated recently that one of the *K. pneumoniae* ST258 clades produces a member of the acyltransferase superfamily 3 (*atf3)* [54]. The acquisition of the *atf3* gene promotes lysine acetylation of multiple central metabolism enzymes, including the glucose-6 phosphate dehydrogenase Zwf. This, in turn, diminishes Zwf activity and enhances glycolysis, tricarboxylic acid (TCA) cycle activity, and ATP generation. The changes in *K. pneumoniae* ST258 acetylome offer an advantage for the pathogen, leading to greater consumption of glucose in the host airway and increased bacterial burden in the lung [54]. Lysine acetylation is also a frequent modification in *A. baumannii* [55]. Proteomic studies revealed that 10% of *A. baumannii* proteins have at least one acetylated lysine residue. The *A. baumannii* acetylome comprises proteins involved in iron uptake systems, biofilm formation, and drug resistance. In *B. subtilis*, lysine acetylation was detected in ~20% of the proteome. Acetylation controlled *B. subtilis* proteins involved in biofilm formation, cell growth, nucleoid compaction, and other essential cellular pathways [62,63,64]. It has been recently reported that the multiple *P. mirabilis* proteins undergo intensive post-translational acylation: acetylation, 2-hydroxyisobutyrylation, and succinylation. Different acyl groups often modify the same lysine residue [15]. Interestingly, all these modifications can be reversed by CobB. It was proposed that CobB deacetylation and de-2-hydroxyisobutyrylation may affect bacterial growth by regulating the catalytic activity of metabolic enzymes, not only in *P. mirabilis* but also in other bacteria [15]. Almost 8000 acetylation sites were found on 1720 *S. aureus* proteins, including ribosomal proteins and enzymes involved in the tricarboxylic acid cycle and glycolysis. The acetylation sites were also frequently succinylated [16], confirming a strong interaction between these two types of modifications in bacteria [15,16].

Results suggesting that lysine acetylation may influence the frequency of persistent bacteria were obtained in this study only in the case of one of the tested clinical isolates: *K. pneumoniae.* Enhanced acetylation resulted in a two-fold increase of *K. pneumoniae* persister levels (Figure 4C). It is evident that, depending on the bacterial species or even clinical isolate, different mechanisms may contribute to persistence. However, certain similarities between acetylation pathways and the homology between KATs and deacetylases in *E. coli* and pathogens suggest that it is worth searching for common factors responsible for the formation of persisters.

## 4. Materials and Methods

### 4.1. Strains and Growth Conditions

*E. coli* BW25113 [F−, Δ(*araD-araB*)567, *lacZ*4787(del)::*rrnB*-3, λ−, *rph-1*, Δ(*rhaD*-*rhaB*), *hsdR*514] was used as a wild-type strain in this study. The BW25113 derivatives deficient in acetylation/deacetylation pathways are presented in Table 3 [24].

To construct the BW25113 Δ*ackA-pta-acs* and Δ*ackA-pta-yfiQ* strains, the Δ*acs* or Δ*yfiQ* mutations were transferred from BW25113 Δ*acs* and BW25113 Δ*yfiQ* to the BW25113 Δ*ackA-pta* strain by P1 transduction. The presence of mutations was verified by PCR using the appropriate primers (Table 4).

The *E. coli* strains were grown at 37 °C for 48 h in TB7 minimal medium supplemented with 0.4% glucose or 25 mM sodium acetate [49]. *Klebsiella pneumoniae ESBL (KPD-577)*, *Acinetobacter baumannii RUH 134*, *Proteus mirabilis* (KPD-452), *Bacillus subtilis* (ATCC 6633), and *Staphylococcus aureus ATCC 25923* were sourced from the Collection of Plasmids and Microorganisms (University of Gdansk) or the American Type Culture Collection (ATCC). The pathogen strains were grown at 37 °C in LB medium supplemented with or without 0.4% glucose.

### 4.2. Isolation and Analysis of Protein Aggregates

*E. coli* protein aggregates were isolated as described previously [24]. Briefly, the bacteria (50 mL culture) were pelleted (10 min, 4000× *g*), suspended in 1 mL of 0.2 M Tris-HCl pH 7.4, and converted into spheroplasts by adding 1 mL of 1 M sucrose in 0.2 M Tris-HCl pH 7.4 and egg-white lysozyme solution (12 mg/mL in 100 mM EDTA pH 7.6) to a final concentration of 60 µg/mL. After 15 min on ice, 2 mL of ice-cold water was added to the suspension. After 15 min, spheroplasts were subjected to sonication in a Vibra-Cell sonicator. After the removal of unbroken cells by centrifugation (15 min, 2000× *g*), the supernatant was incubated with 2% of Triton X-100 for 15 min at room temperature. Insoluble aggregates were pelleted after 30 min of centrifugation at 21,000× *g* and washed twice with 50 mM Tris-HCl pH 7.4. Protein aggregates and whole-cell extracts were resolved by SDS-PAGE and analyzed using ImageJ version 1.53k to estimate the amount of aggregated proteins in relation to the total protein content in whole-cell extracts (set to 100%).

### 4.3. Immunodetection of Acetyl-Lysine

SDS-PAGE and Western blotting were performed according to the standard procedures. Acetylated proteins were immunodetected using anti-acetyl-lysine antibodies (Abcam, Cambridge, UK), anti-rabbit peroxidase conjugate (Sigma, St. Louis, MO, USA), and Clarity Western ECL Substrate (Bio-Rad, Hercules, CA, USA). The immunoblots were scanned using the Azure 500 imaging system and quantified using ImageJ.

### 4.4. Determination of the Number of Persister, Nonculturable, and Dead Cells

To determine the number of persisters, the cultures were diluted 1:100 in fresh TB7 or LB medium, supplemented with appropriate antibiotics (at a concentration of 50 × MIC): ofloxacin (*E. coli* cultures) and meropenem (*A. baumannii*, *K. pneumoniae*, *P. mirabilis).* The cultures were incubated at 37 °C for at least 6 h, until the killing curves reached a biphasic plateau. The surviving persisters were plated on LB agar for colony counts. To rule out the possibility that antibiotic-tolerant cells were resistant mutants, the colonies were plated on LB agar supplemented with an appropriate antibiotic. The number of persisters was estimated relative to the total number of CFU before antibiotic treatments. The total cell number was determined using a Neubauer chamber at 1000-fold magnification. The amount of dead cells was estimated using an epifluorescence microscope (Axio Scope.A1, Zeiss, Oberkochen, Germany) after staining with a LIVE/DEAD BacLight viability kit (Molecular Probes, Eugene, OR, USA) according to the manufacturer’s protocol. Nonculturable propidium iodate (PI)-excluding cells are often referred to as VBNC (viable but nonculturable) bacteria. However, other authors suggest that VBNC cells are dead despite having intact membranes [65]. Therefore, we classified bacterial cells as culturable (able to form colonies and PI-negative), nonculturable (PI-negative, which corresponds to VBNC cells), or dead (PI-positive) bacteria. The number of nonculturable cells was calculated by subtracting CFU counts from the number of live cells; CFU was estimated by plating serial dilutions on LB agar plates.

## Figures and Tables

**Table 1:** *E. coli* mutants deficient in acetylation and deacetylation pathways (see Figure 1).

Strain	Lack of
Δ*ackA*	Acetate kinase (EC 2.7.2.1)
Δ*pta*	Phosphate acetyltransferase (EC 2.3.1.8)
Δ*ackA-pta*	Acetate kinase (EC 2.7.2.1) and phosphate acetyltransferase (EC 2.3.1.8)
Δ*acs*	Acetate–CoA ligase (EC 6.2.1.1)
Δ*yfiQ*	Protein lysine acetyltransferase (EC 2.3.1.48)
Δ*cobB*	Protein lysine deacetylase (EC 2.3.1.286)
Δ*ackA-pta-acs*	Acetate kinase (EC 2.7.2.1), phosphate acetyltransferase (EC 2.3.1.8), and acetate–CoA ligase (EC 6.2.1.1)
Δ*ackA-pta-yfiQ*	Acetate kinase (EC 2.7.2.1), phosphate acetyltransferase (EC 2.3.1.8), and protein lysine deacetylase (EC 2.3.1.286)

**Table 2:** Percentage of persister, nonculturable, and dead cells in *E. coli* cultures after ofloxacin treatment. The maximum numbers of persistent and nonculturable bacteria are boldfaced.

	WT	Δ*ackA*	Δ*pta*	Δ*ackA-pta*	Δ*acs*	Δ*yfiQ*	Δ*cobB*	Δ*ackA-pta-acs*	Δ*ackA-pta-yfiQ*
+glucose									
Persisters (culturable)	0.1 ± 0.01	**2.7** ± 0.16	0.8 ± 0.16	1.3 ± 0.25	0.1 ± 0.04	0.03 ± 0.01	0.4 ± 0.14	0.03 ± 0.01	0.03 ± 0.01
Nonculturable	60 ± 3.3	**82** ± 5.4	64 ± 2.4	62 ± 5.4	60 ± 6.2	48 ± 5	55 ± 3.7	35 ± 7	33 ± 4
Dead	40 ± 3.3	15 ± 5.5	35 ± 2.4	37± 5.5	40 ± 6.2	52 ± 5	45 ± 3.8	65 ± 7	67 ± 4
+acetate									
Persisters (culturable)	0.5 ± 0.14	0.8 ± 0.37	**6.0** ± 0.44	3.9 ± 0.19	0.1 ± 0.02	0.1 ± 0.01	0.6 ± 0.1	0.06 ± 0.01	0.04 ± 0.01
Nonculturable	56 ± 3.7	49 ± 3.6	**86** ± 0.5	66 ± 3.7	63 ± 4.2	50 ± 5	55 ± 6.3	33 ± 4.4	36 ± 5
Dead	44 ± 3.8	50 ± 2.2	8 ± 0.44	30 ± 3.6	37 ± 4.1	50 ± 5	44 ± 6.2	67 ± 4.4	64 ± 5

**Table 3:** *E. coli* BW25223 mutants.

Strain	Source *	Mutation
Δ*ackA*	CGSC#:9834 (JW2293-1)	*ackA*-778(del)::kan
Δ*pta*	CGSC#:9844 (JW2294-1)	*pta*-779(del)::kan
Δ*ackA-pta*	[24]	*ackA-pta* (del)::cat
Δ*acs*	CGSC#:10898 (JW4030-1)	*acs*-763(del)::kan
Δ*yfiQ*	CGSC#:10042 (JW2568-1)	*yfiQ*-752(del)::kan
Δ*cobB*	CGSC#:9039 (JW1106-1)	*cobB*-779(del)::kan
Δ*ackA-pta-acs*	This study	*ackA-pta* (del)::cat, *acs*-763(del)::kan
Δ*ackA-pta-yfiQ*	This study	*ackA-pta* (del)::cat, *yfiQ*-752(del)::kan

* KEIO Collection number in brackets.

**Table 4:** Primers used to verify the presence of mutations.

Primer	Sequence 5′ → 3′	
pta-F	GCTCAGCTGGCGGTGCTGTTTTGTAAC	Amplification of the *pta* gene
pta-R	CAAAGCTGCGGATGATGACGAGATTAC	
ack-F	CATCATGCGCTACGCTCTATGGCTCC	Amplification of the *ackA* gene
ack-R	AGCTGAGCTGGCGGTGTGAAATCAG	
acs-F	AAACCGTTACCGACTCGCAT	Amplification of the *acs* gene fragment
acs-R	ACCCTGCCGTTTATTTGCAC	
yfiQ-F	TCTGGCAGGAAAAACGCAAC	Amplification of the *yfiQ* gene fragment
yfiQ-R	ATGCAGACGACATAAGCGGG	
cob-F	GTGCGGCCTTCCTACATCTAA	Amplification of the *cobB* gene fragment
cob-R	TGCCGCATTGTTATTGACGAG	
ack-F	CATCATGCGCTACGCTCTATGGCTCC	Amplification of the *ackA-pta* DNA fragment
pta-R	CAAAGCTGCGGATGATGACGAGATTAC	

F—forward, R—reverse.

## Data Availability

Data are contained within the article and supplementary materials.

## Supplementary Materials

The following supporting information can be downloaded at https://www.mdpi.com/article/10.3390/molecules29020383/s1: Table S1: Levels of aggregates, acetylated proteins, and viable cells in *E. coli* strains with impaired acetylation/deacetylation pathways.
