# Supplementary material for: Influence of Nε-Lysine Acetylation on the Formation of Protein Aggregates and Antibiotic Persistence in E. coli
Source: Molecules. 2024 Jan 12;29(2):383. doi: 10.3390/molecules29020383 (PMC10819833; doi:10.3390/molecules29020383)
Supplement: Supplementary file 1 [file molecules-29-00383-s001.zip › molecules-2810629-supplementary.pdf]

Table S1. Levels of aggregates, acetylated proteins and viable cells in *E. coli* strains with impaired acetylation/deacetylation pathways. The bacteria were grown in TB7 medium supplemented with 0.4% glucose or 25 mM acetate.

	Strains	Aggregates (%)	Relative level of acetylation	Viable cells (%)
+ glucose	WT	5.0 ± 0.50	1.0 ± 0.09	65 ± 5
	Δ*ackA*	7.3 ± 0.78	2.1 ± 0.20	98 ± 2
	Δ*pta*	5.1 ± 0.51	0.4 ± 0.04	75 ± 6
	Δ*ackA-pta*	5.5 ± 0.57	0.2 ± 0.03	75 ± 7
	Δ*acs*	3.9 ± 0.37	0.6 ± 0.06	60 ± 5
	Δ*yfiQ*	4.1 ± 0.38	0.3 ± 0.02	67 ± 6
	Δ*cobB*	5.2 ± 0.54	2.7 ± 0.22	60 ± 6
	Δ*ackA-pta-acs*	2.9 ± 0.24	0.01 ± 0.002	55 ± 4
	Δ*ackA-pta-yfiQ*	3.2 ± 0.28	0.1 ± 0.01	55 ± 5
+ acetate	WT	7.0 ± 0.64	3.7 ± 0.04	60 ±4
	Δ*ackA*	7.0 ± 0.55	3.7 ± 0.39	70 ±6
	Δ*pta*	9.2 ± 0.53	4.4 ± 0.51	95 ±4
	Δ*ackA-pta*	7.5 ± 0.70	2.9 ± 0.32	80 ± 4
	Δ*acs*	6.1 ± 0.80	3.4 ± 0.37	81 ± 6
	Δ*yfiQ*	6.4 ± 0.63	3.0 ± 0.32	60 ± 5
	Δ*cobB*	7.2 ±0.60	3.9 ± 0.41	70 ± 6
	Δ*ackA-pta-acs*	5.1 ± 0.76	0.2 ± 0.02	65 ± 5
	Δ*ackA-pta-yfiQ*	4.9 ± 0.50	0.2 ± 0.03	65 ± 5
